# Mobile nucleic acid amplification testing (mobiNAAT) for Chlamydia trachomatis screening in hospital emergency department settings

**DOI:** 10.1038/s41598-017-04781-8

**Published:** 2017-07-03

**Authors:** D. J. Shin, P. Athamanolap, L. Chen, J. Hardick, M. Lewis, Y. H. Hsieh, R. E. Rothman, C. A. Gaydos, T. H. Wang

**Affiliations:** 1 Department of Biomedical Engineering, The Johns Hopkins University, Baltimore, MD 21218 USA; 2 Department of Mechanical Engineering, The Johns Hopkins University, Baltimore, MD 21218 USA; 3 Division of Infectious Diseases, School of Medicine, The Johns Hopkins University, Baltimore, MD 21218 USA; 4 Department of Emergency Medicine, School of Medicine, The Johns Hopkins University, Baltimore, MD 21218 USA; 5 Institute for NanoBioTechnology, The Johns Hopkins University, Baltimore, MD 21218 USA

## Abstract

Management of curable sexually-transmitted infections (STI) such as Chlamydia can be revolutionized by highly sensitive nucleic acid testing that is deployable at the point-of-care (POC). Here we report the development of a mobile nucleic acid amplification testing (mobiNAAT) platform utilizing a mobile phone and droplet magnetofluidics to deliver NAAT in a portable and accessible format. By using magnetic particles as a mobile substrate for nucleic acid capture and transport, fluid handling is reduced to particle translocation on a simple magnetofluidic cartridge assembled with reagents for nucleic acid purification and amplification. A mobile phone user interface operating in tandem with a portable Bluetooth-enabled cartridge-processing unit facilitates process integration. We tested 30 potentially *Chlamydia trachomatis* (CT)-infected patients in a hospital emergency department and confirmed that mobiNAAT showed 100% concordance with laboratory-based NAAT. Concurrent evaluation by a nontechnical study coordinator who received brief training via an embedded mobile app module demonstrated ease of use and reproducibility of the platform. This work demonstrates the potential of mobile nucleic acid testing in bridging the diagnostic gap between centralized laboratories and hospital emergency departments.

## Introduction

Curable sexually transmitted infections still present a major concern for public health. Among them, *Chlamydia trachomatis* (CT) is one of the most common infectious diseases in the United States^1^. Because of the high prevalence of asymptomatic infection^2^ and serious complications associated with missed infections, including infertility and ectopic pregnancy^3^, routine screening of all sexually active women under the age 26 years is now recommended as the standard of care^4^. Unfortunately, only 38% of potential patients actually receive screening, which is in large part due to a significant deficiency in sensitivity and accessibility for the commercially available chlamydia tests^5^. For example, lateral-flow device (LFD) rapid tests based on immunochromatography are easy to use and widely available, but they suffer from alarmingly poor sensitivity at 41–87%^6–8^. In contrast, nucleic acid amplification tests (NAATs) are the de facto gold standard assays recommended by the Centers for Disease Control and Prevention (CDC) with >90% clinical sensitivity and 99% specificity^9, 10^. However, NAATs remain tied to the clinical laboratory because of the technical challenges associated with implementing the conventional bioassay workflow such as multi-step fluidic processing in a simple and affordable format. Comparative studies suggest that point-of-care (POC) tests that are both sensitive and affordable may substantially change the discourse of chlamydia surveillance^10, 11^, highlighting a major unmet need for a platform technology capable of delivering laboratory-based assays in a POC setting.

**Figure 1:**
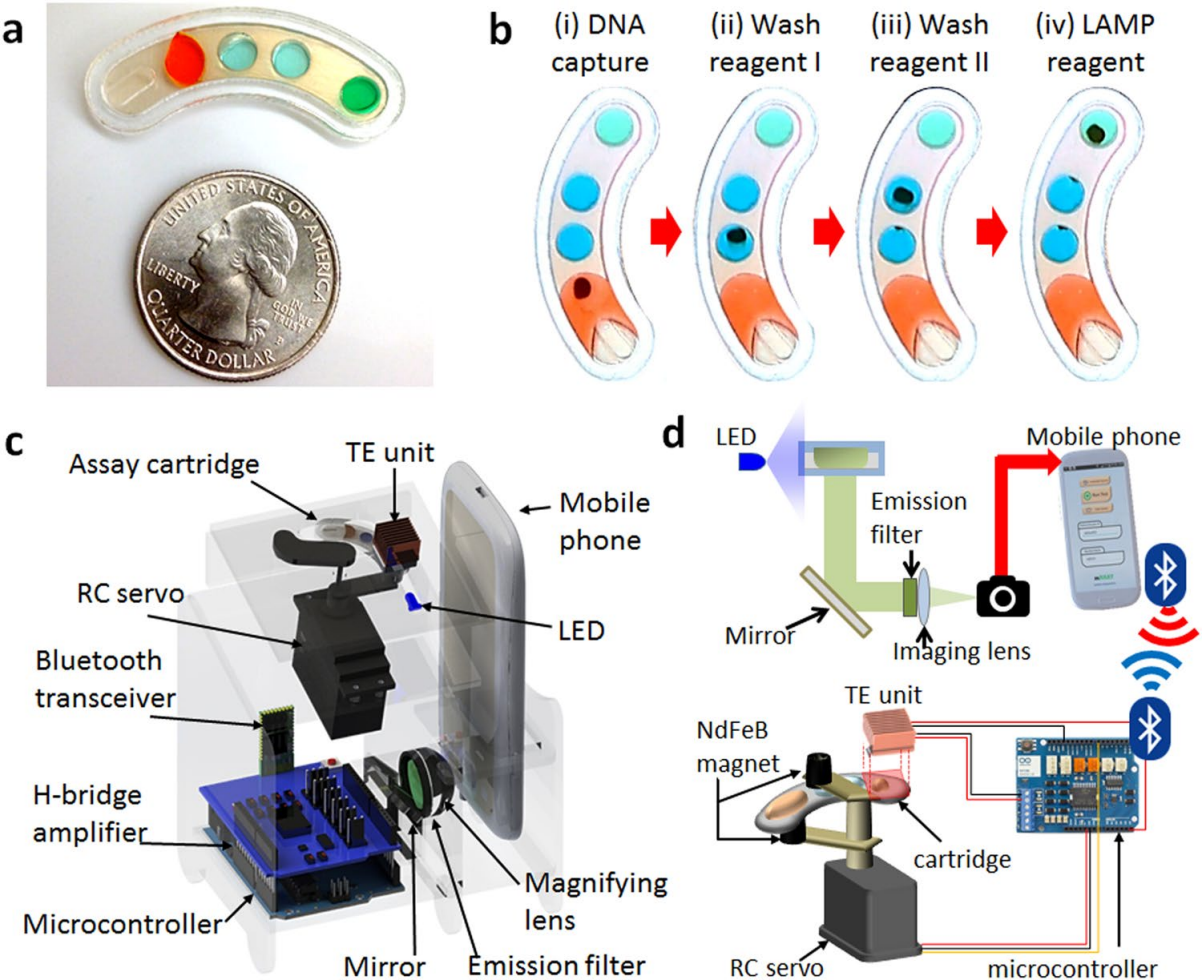
Technology overview. (**a**) Photograph of droplet magnetofluidic cartridge next to US quarter for comparison. (**b**) Time-lapse sequence of particle transport on cartridge. DNA is captured on the magnetic particle surface in (i), followed by successive rinsing steps in wash buffers (ii) and (iii), and subsequent elution and amplification in LAMP reagent (iv). (**c**) CAD layout of components inside the cartridge-processing unit. The unit integrates several subsystems to facilitate mechanical manipulation for magnetic particle handling, thermal control for assay initiation and optical signal acquisition with the assistance of a mobile phone. (**d**) Functional overview of instrumentation. The mobile phone interacts with the instrument in two ways: first, the embedded CMOS camera sensor is used to collect signal via an optical signal relay chain, in which the LED illuminates the reaction chamber to generate fluorescent signal that is relayed by a mirror and filtered prior to being magnified by a combination of an external imaging lens and an embedded camera lens on the phone before reaching the sensor. Secondly, the mobile phone facilitates wireless communication with the instrument’s on-board microcontroller to coordinate magnetic particle transport and thermal incubation.

Here we present a standalone, sample-to-answer mobile NAAT system for chlamydia surveillance, incorporating a droplet magnetofluidic cartridge processed in tandem with a portable electromechanical unit operated by a mobile phone interface. Analyte capture and transport via magnetofluidic manipulation replace conventional fluidic operations and the supporting instrumentation, substantially reducing the complexity of platform design and operation. The cartridge has a dimension comparable to a USB stick (Fig. 1a) and a material cost of less than $2, including all reagents, comparable to today’s lateral-flow devices. Meanwhile, the cartridge-processing unit is less than $200 (Table S1), suggesting a hundredfold reduction in cost as compared to existing platforms. Central to the platform design is the mobile phone which enables the user to coordinate the various critical operations of the assay including magnetic actuation, thermal incubation, and signal acquisition and processing. Further assisted by a video-based training module embedded in the mobile app, we developed an easily accessible NAAT platform for trained and naïve operators alike. The mobiNAAT platform demonstrates high analytical sensitivity and specificity for urogenital chlamydia, as well as clinical sensitivity on par with laboratory-based NAAT platforms. The streamlined user interface and training module allow an unexperienced operator to obtain accurate clinical results in approximately 1 hour.

## Results

### Platform design principle

From an engineering perspective, designing a POC platform presents a dual challenge. The first challenge is a mechanical problem of assay integration, while the second challenge is that of process integration in a manner that is accessible to the end user. Here, we identified a solution to providing simple fluidic integration via droplet magnetofluidics and enhancing platform accessibility via mobile phone technology.

The conventional nucleic acid testing workflow involves an iterative process of addition and removal of aqueous reagents for the capture and purification of nucleic acids on a solid substrate (e.g., magnetic particles), followed by the elution of purified sample for analysis using an enzymatic amplification reaction such as polymerase chain reaction (PCR). Though simple in principle, the manual process for transporting and fluidic handling is repetitive and laborious. More critically, conventional approaches to miniaturize and automate such processes involve complex device designs and control systems for routing and valving the fluid, thus resulting in generally expensive and bulky platforms^12–14^. It is highly desirable to develop a simpler and more scalable fluidic handling strategy to address the need for POC utilizations. To that end, we designed a fluidic workflow based on the principles of droplet magnetofluidics^15, 16^ (Fig. 1a). In this workflow, fluidic manifolds are replaced by a series of discrete, stationary droplet reservoirs interconnected by a smooth hydrophobic surface. Analyte capture and transport are facilitated by surface-modified magnetic particles, which can traverse between each reservoir when acted upon by an external magnetic field (Fig. 1b). This approach obviates the need for bulk fluid transport, and it circumvents typical design constraints associated with fluidic routing, valving, measuring and dead volume. As a result, the disposable cartridge retains a simple fabrication workflow and has resilience to conventional modes of device failure such as clogging and leakage.

Using magnetofluidics as the foundation of our approach, we developed a system in which the droplet cartridge is processed in a streamlined fashion using a mobile phone in tandem with a cartridge-processing unit (Fig. 1c). The mobile phone has recently been noted for its potential utility in biomedical applications because of its ability to harness the embedded CMOS optical sensor^17–20^, its capacity for networking^21–23^, and its capacity for data-processing^24^, although a full-scale integration of complex molecular assays such as NAAT has not yet been reported. In our design, we combined various elements of the mobile phone hardware to miniaturize and streamline the workflow of a sample-to-answer NAAT assay. In addition to optical signal acquisition and an app-based user interface, Bluetooth connectivity enables us to extend the mobile phone’s capability to include non-native functions such as mechanical and thermal actuation. With the Bluetooth-enabled cartridge-processing unit, we can automate and coordinate magnetofluidic actuation and isothermal amplification, facilitating complete process integration from sample lysate to detection.

### Assay workflow design for mobiNAAT platform

The mobiNAAT platform workflow begins with a two-step sample loading, where the sample matrix presented in the form of a vaginal swab is initially expressed in a tube containing lysis reagent for cell lysis. When the expressed sample is transferred from the tube into the cartridge using a syringe, the sample can be further lysed by attaching a syringe-linked bead-beating module^25^ for mechanical disruption of remaining cells. With the sample loaded, the cartridge is inserted into the cartridge-processing unit. The operator is assisted by a checklist on the mobile app to ensure that the manual steps have been performed correctly. Each step of the assay is clarified further by the inclusion of a video instruction embedded in the app (Video S1). Once all of the manual steps are completed in approximately 2 minutes, the mobile phone is docked into the cartridge-processing unit, and automated cartridge processing is initiated.

**Figure 2:**
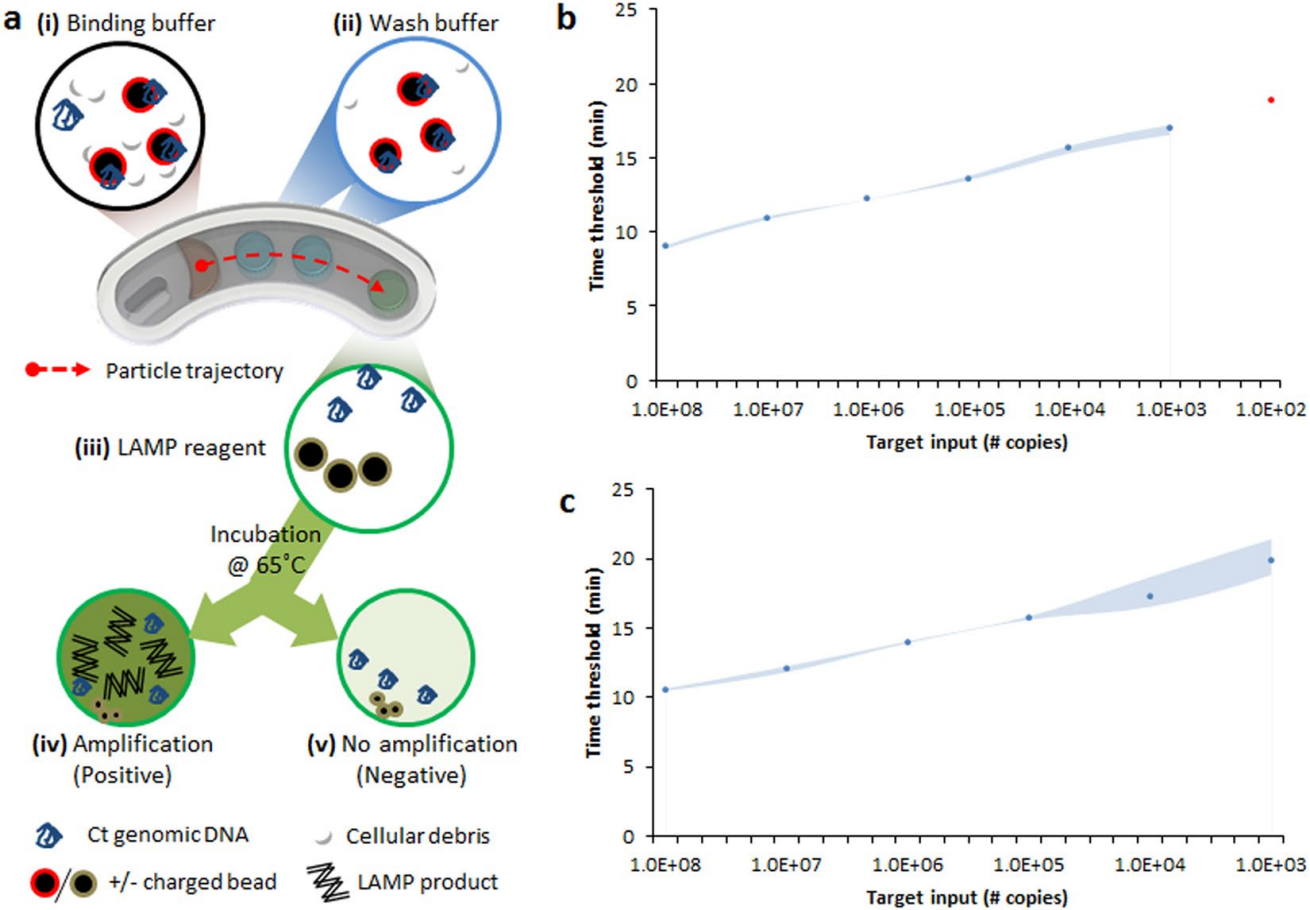
Assay design and sensitivity characterization. (**a**) Schematic of single-stream LAMP assay as implemented on the mobiNAAT platform. Magnetic particles capture nucleic acid targets from sample lysate via electrostatic interaction, where the acidic pH maintained by the binding buffer (i) generates a positive charge on the polyhistidine-coated bead surface. Affinity between particles and nucleic acids is maintained at acidic pH (<pH 5) of the wash buffers (ii), which is reversed upon entry in the LAMP amplification mixture (iii) with a basic pH of 8.5. Subsequent amplification (iv) generates a green fluorescent signal based on the complexometric indicator calcein, contrasting with unamplified reagent (v). (**b**) Comparison among amplification of dilutions of synthetic DNA spiked into reaction mixture. A fraction of the replicate reactions were amplified for 10^2^ copies of molecular target input (red marker, 1/3), indicating analytical sensitivity of 10^2^–10^3^ copies of molecular target. Shaded areas represent upper and lower bounds of time threshold (n = 3). (**c**) Evaluation of magnetic particle-based DNA capture, elution and subsequent amplification as a function of DNA quantity in lysis/binding buffer. Time thresholds obtained indicate sensitivity and process efficiency that are comparable to the standard LAMP process without bead coupling. Shaded areas represent upper and lower bounds of time threshold (n = 3).

We developed a magnetofluidics-assisted loop-mediated isothermal amplification (LAMP) assay for nucleic acid target amplification^26^ as shown in Fig. 2a. This approach combines nucleic acid extraction and purification with amplification by direct elution of target nucleic acids into the amplification reagent. Assay sensitivity was characterized and found to be sensitive to 10^2^–10^3^ copies of molecular target (Fig. 2b), which is suitable for chlamydia samples collected from vaginal swabs^27^. Assay specificity was characterized against a panel of genomic DNA obtained from endogenous and pathogenic vaginal flora, and the absence of crosstalk was verified (Fig. S1).

Sample is processed in the cartridge as follows. First, magnetic particles capture nucleic acid targets from sample lysate via electrostatic interaction, where the acidic pH maintained by the binding buffer sustains a positive charge on the polyhistidine-coated bead surface. Affinity between particles and nucleic acids is maintained at acidic pH (<pH 5) of the wash buffers, which is reversed upon entry in a LAMP amplification mixture. LAMP-based amplification strategy is highly amenable to the mobiNAAT platform for several important reasons. First, isothermal amplification assay greatly relaxes thermal conductivity constraints of the cartridge, allowing flexibility in cartridge material selection and fabrication strategy. Second, the capacity of LAMP assay to generate a superabundance of pyrophosphate enabled us to couple these reactions to colorimetric or fluorescent indicators^28, 29^, generating signals that can be analyzed objectively with a mobile phone CMOS sensor. Lastly, the pH of the LAMP reaction condition was readily compatible with the elution conditions for nucleic acid purification chemistry, allowing seamless transition from nucleic acid extraction to target amplification using the same solid phase transport. We utilized calcein as an indicator dye into the amplification mixture to enable fluorescent assessment of amplification using the mobile phone CMOS sensor^28, 29^.

### Using the mobiNAAT platform to process clinical specimen

**Figure 3:**
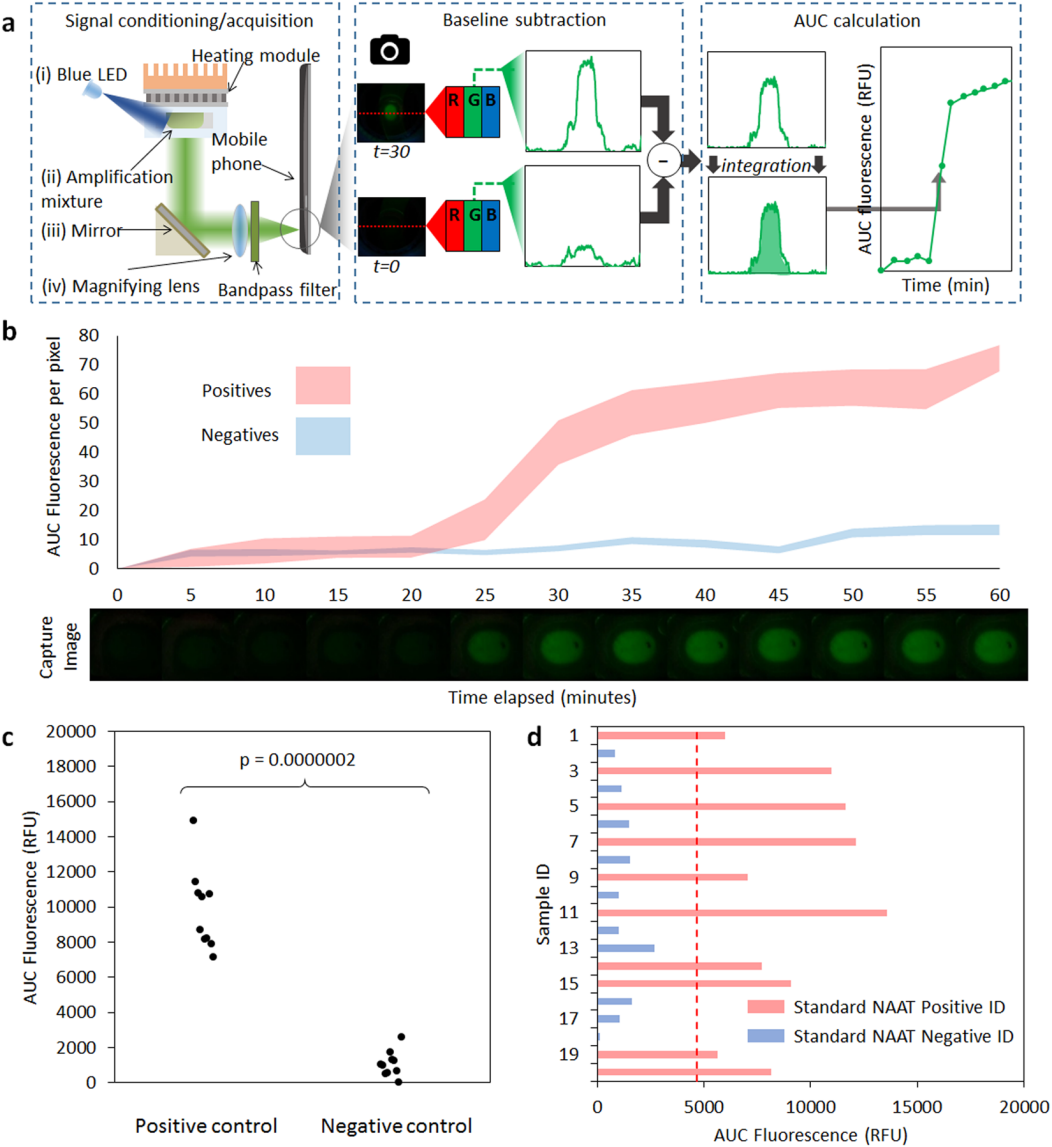
Signal acquisition and platform validation study. (**a**) Image processing algorithm on the mobile phone. Initially, image is acquired at the start of the incubation (time = 0). Signal acquisition is performed using an optical pre-processing signal relay chain as described in Fig. 1c. Subsequent images are captured and segmented to extract information from a single row of pixels across the incubation chamber. Only the green value of the RGB data is used. Data acquired at each time point are normalized by subtracting segmented data from time = 0. Afterwards, the area under the resultant vector is integrated in order to yield a single value, which is used either for end-point analysis or plotted sequentially over time for real-time monitoring. (**b**) Real-time monitoring of select deblinded clinical samples using time-lapse image acquisition. Positive samples show development of signal and are clearly differentiated from signal developed by negative samples beginning at approximately 20 minutes of incubation. Shaded areas represent upper and lower bounds of AUC fluorescence (n = 3). Bottom panel shows image of fluorescence developing over time in a positive control reaction in droplet cartridge. (**c**) Reproducibility test showing signal generated from chlamydia DNA target-positive and negative cartridges (n = 10 each). Welch’s two sample t-test yields p < 0.000001. (**d**) Validation of the mobiNAAT platform using blinded vaginal swab samples obtained from an equal number of chlamydia-positive and -negative individuals (n = 20). AUC fluorescence levels of swab samples that are positive (blue) or negative (orange) are shown. Welch’s two sample t-test yields p < 0.00001. Classification threshold (red dotted line) is established using signals obtained from a set of control samples (n = 10).

Once the LAMP assay was characterized, we proceeded to implementing the assay on the cartridge. In order to interpret the raw fluorescence signal generated via amplification reaction, we first developed an image-processing algorithm as described in Fig. 3a. In brief, an image of the reaction chamber was captured using the mobile phone camera at the start of the incubation period and at user-specified intervals thereafter. Once an image was acquired, a single vertical line of pixels was sampled from the green component of RGB values for each image at the center of the reaction chamber at a preset image coordinate. Vectors representing each time point were subtracted by the baseline vector acquired at the start of the incubation and integrated in order to obtain the area under the curve (AUC) fluorescence corresponding to each time point. A representative image of fluorescence signal that develops over the course of a positive reaction is presented in Fig. 3b.

Next, we tested our platform using a set of clinical samples in order to assess the platform’s capacity to differentiate between positive and negative samples. Further analysis of the samples using real-time trace (Fig. 3b) showed that a fluorescence signal began to develop consistently after 20 minutes of incubation, which subsequently was used as the initial time point for image analysis. Reproducibility of fluorescence measurement on cartridge was tested using 20 cartridges loaded with a training set of 10 positive and 10 negative reactions (Fig. 3c). We then evaluated the performance of the cartridge by analyzing archived vaginal swab samples from 20 individuals obtained from the Johns Hopkins Center for Point of Care Tests for Sexually Transmitted Diseases. One set of swabs was analyzed using the Gen-Probe Aptima Combo 2 assay. The superb analytical performance of the Gen-Probe assay makes this laboratory-based NAAT the current standard of care in *Chlamydia trachomatis* diagnostics^30^. The second set of swabs was tested on the mobiNAAT platform. The two results were in agreement for 20 out of 20 samples, demonstrating that the mobiNAAT performance is comparable to standard clinical laboratory-based NAAT assay performance for the samples tested (Fig. 3d).

### Evaluation of mobiNAAT platform in emergency care setting

**Figure 4:**
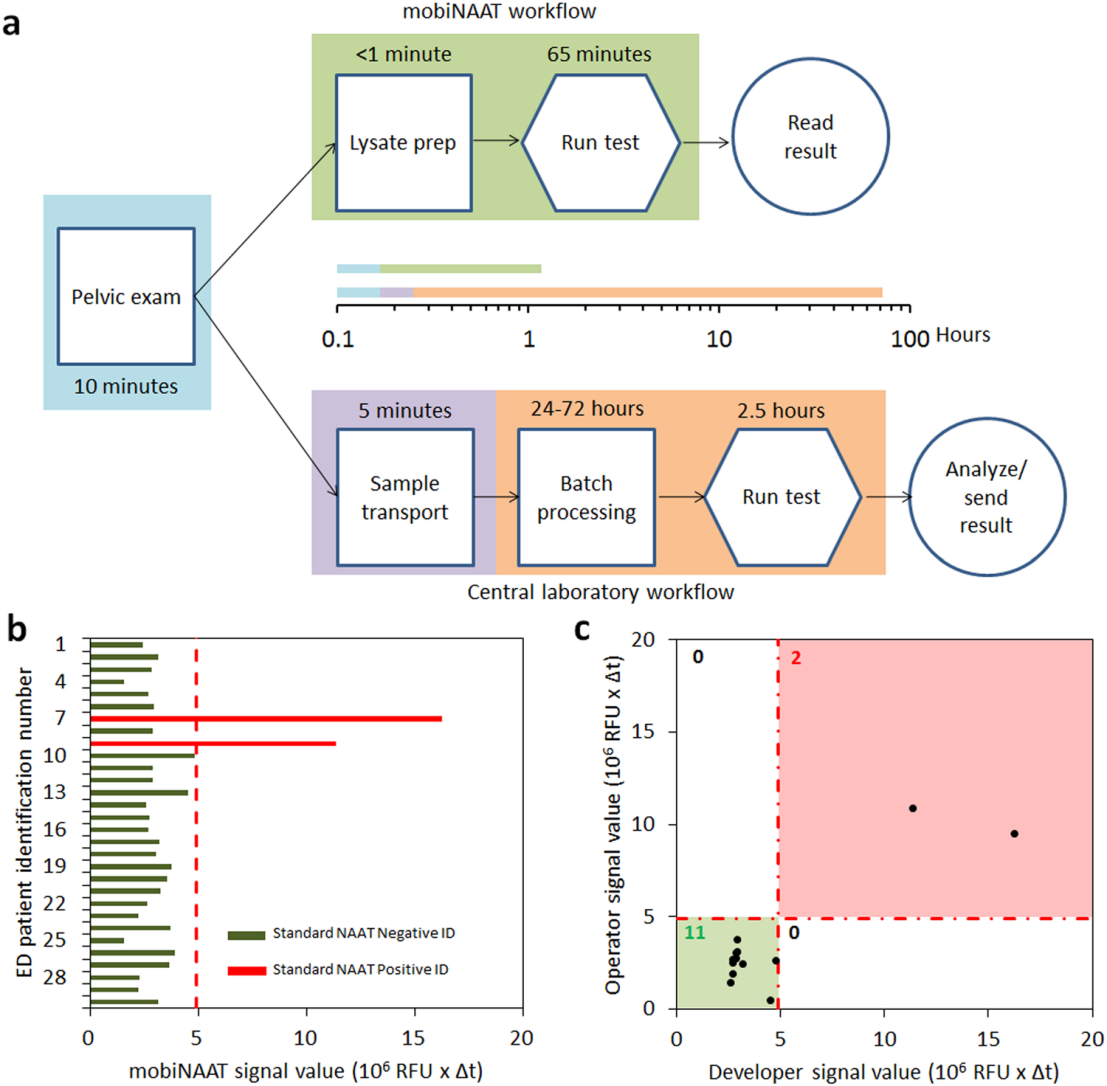
Platform evaluation at ED. (**a**) Comparison of standard and mobiNAAT workflows in the emergency room. In a standard workflow, the vaginal swab acquired during a pelvic exam by the provider is collected by a study coordinator, who transports the sample to the microbiology laboratory. The sample is then barcoded and batch processed to be tested using the standard-of-care NAAT assay. Afterwards, the test result is made available on the electronic medical record (EMR) accessible by the provider. In the mobiNAAT workflow, the test is performed directly in the emergency room via workflow as described in Fig. 2a, and the result is made available directly on the mobile device. Standard approach via central laboratory yielded >72 hours due to downtime inherent to batch processing, whereas mobiNAAT-enabled testing in the emergency room yields diagnostic results in approximately 1 hour. (**b**) Comparison of results obtained using the mobiNAAT platform using clinical samples collected at the emergency room (n = 30). Samples were tested in parallel using the Gen-Probe Aptima Combo 2 assay for verification (positives marked by red bars; negatives marked by green bars; instrument data in Fig. S8). Among all patient samples tested, two patients (ID 7 and 9) reported positive in the verification assay, in agreement with the result obtained using the mobiNAAT platform. Classification threshold (red dotted line) is established using signals obtained from a set of control negative samples (n = 15) (Fig. S9). (**c**) Comparison of results obtained using mobiNAAT platform by the developers and a naïve operator trained using the mobile app (n = 13). Patients 5–17 were tested in parallel by a clinical staff member at the emergency room (green markers) and showed full correspondence with the results obtained by the developers (11 negative, 2 positive).

In order to evaluate the performance of the mobiNAAT platform in a clinical setting, we delivered the mobile phone pre-loaded with the app software, the cartridge-processing module, and the assembled cartridges to the Emergency Department at Johns Hopkins Hospital (Baltimore, MD, USA). Volunteers for the study were consented and recruited from a set of patients visiting for pelvic examination in the emergency department. Samples from each patient were tested in parallel by the mobiNAAT platform (Fig. 4a) and the Gen-Probe Aptima Combo 2 assay for verification (Fig. S8). The entire mobiNAAT workflow included six minutes of user training for the first-time operators, minimal hands-on time for sample loading, and 65 minutes of automated cartridge processing. The mobiNAAT platform correctly identified two positives among 30 patients, once again, demonstrating that the droplet assay performance is comparable to the gold standard performance for the samples tested (Fig. 4b).

In order to assess the accessibility of the platform to a context-relevant end user, an on-site nontechnical research coordinator using the platform for the first time (naïve operator) was trained using the training modules accessed via the mobile app. Subsequent comparison of results obtained from the app-trained naïve operator and platform developers showed full correspondence for the samples tested, demonstrating the ease of use facilitated by the mobile phone interface and the training module (Fig. 4c).

## Discussion

STIs represent a significant public health burden in the United States because of the high morbidity and mortality and because of the associated costs. Numerous factors contribute to the persistent STI public health burden, but of particular importance is the delay in treatment resulting from lengthy diagnostic protocols. Comparative effectiveness studies attest to the importance of cost and sensitivity as decisive parameters for patients to seek POC tests over standard laboratory-based NAAT^11^. An affordable and accessible NAAT platform as demonstrated in this work has the potential to change the diagnostic workflow for patients in numerous emergency settings. In particular, POC tests offer important strategies to address the chlamydia epidemic, because diagnosis and immediate treatment can prevent transmission to sexual partners and associated sequela.

POC tests based on immunoassay have gathered criticism due to poor clinical sensitivity^6, 7^. A direct comparison of clinical sensitivity for various CT assays using endocervical swab specimens showed <40% sensitivity for LFD tests compared to near 100% for NAAT^31^. These studies reinforced our interest in designing an engineering solution to implement NAAT in a format that is affordable, reproducible, and easy to use. The relevant range of chlamydia load from vaginal swabs is 5,167–21,163 elementary bodies per swab with 95% confidence interval^27^, which is in abundance by more than an order of magnitude greater than the analytical sensitivity range of 10^2^–10^3^ copies of molecular target per sample as described in this work.

Rapid delivery of results is an important consideration for POC testing to have an impact in the clinical pathway. A recent study involving 1,356 patients showed 50% of participants unwilling to wait longer than 40 minutes for a POC test result^32^. In the current LAMP-based assay, we have observed amplification signal reach saturation in the majority of cases in approximately 40 minutes of incubation, suggesting an optimized workflow of approximately 50 minutes from start to finish. Since magnetofluidic manipulation takes less than 5 minutes, incubation time is the greatest source of delay in the current workflow. We envision the possibility of extending our approach to faster NAATs to yield a shorter time to obtain actionable diagnostic results. Recombinase polymerase amplification (RPA) is a promising choice with reported threshold times of less than 10 minutes for amplification^33^.

The real-time amplification data processing capability also highlights a possible application of the mobiNAAT platform in quantitative nucleic acid-based testing. LAMP assay is amenable to quantitative measurement based on amplification time until saturation^34^. This is especially pertinent in the field of companion diagnostics, in which the patient’s response to treatment such as antiviral drugs is monitored by measuring the changes in HIV or HCV viral loads^35, 36^. Furthermore, the platform’s compatibility with real-time signal acquisition renders it flexible to strategies for multiplexing via broad-based pathogen identification techniques based on molecular beacons and melting curve analysis^37, 38^.

The use of mobile phone as an integral component of a diagnostic system presents an opportunity to enhance the accessibility of complex operations in a clinical setting. However, there are two practical challenges to this approach. Firstly, the frequency at which mobile phones and their components are updated by the industry makes it challenging to prevent obsolescence of platforms designed around this device. While a modular adapter component analogous to phone cases could be envisioned to accommodate changes in phone dimensions and alignment, mechanical solutions cannot overcome variability in optical sensors. Recent efforts suggest that inter-device calibration using a set of reference images is a feasible approach to overcome this barrier^18, 39^. A second challenge is associated with security concerns over patient data stored on mobile devices. While the management of test results was abstracted to a simple on-device data archive in the scope of this work, additional provisions are required to securely manage protected health information (PHI). As conventional de-identification method does not fully address security concerns for patient test data, it would be prudent to implement organization-wide network security measures for all mobile devices including encryption, firewalls, security software and deactivation of file-sharing services^40^.

A long-term goal envisioned by this research is to develop a versatile and affordable NAAT platform that can be operated by an untrained person in the absence of laboratory infrastructure. The ASSURED guideline for POC tests set by the World Health Organization (WHO) includes affordability, operability by untrained person, and delivery to end users as its criteria^41^, which were the factors that we focused on implementing in this platform. A major strength of this platform is the ability to perform conventional assays at a substantially reduced cost and complexity to the user, so we find this technology to be most applicable to meeting diagnostic needs for underserved clients in today’s health care systems. Future technological milestones toward this direction include independence from cold chain transport and storage for deployment in resource-limited settings, as well as a further streamlined workflow for the initial sample loading into the platform.

## Materials and Methods

### Design and fabrication of the mobiNAAT cartridge-processing unit and software

Computer-assisted drawing (CAD) software (Solidworks 2013, Dassault Systemes SOLIDWORKS, Waltham, MA, USA) was used to construct a prototype enclosure that incorporates an Arduino Uno R3 microcontroller and motor shield, a rotary actuator (Hitec RCD, Porway, CA, USA), thermal incubators (Custom Thermoelectric, Bishopville, MD, USA) and optical components (Thorlabs, Inc., Newton, NJ, USA). All components were designed and assembled using PMMA sheets and acrylic adhesive solvent (WeldOn Adhesives, Inc., Compton, CA, USA) where necessary. A pair of cylindrical NdFeB magnets (6-mm diameter) was affixed on each of the spoke tips to provide permanent magnetic fields for particle manipulation.

The software for the mobile app was developed using Android Studio (Google, Inc., Mountain View, CA, USA). In relation to the processing module, the app operates as a serial communicator that initiates various pre-programmed routines on the microcontroller via Bluetooth communication. The mobile app (Fig. S2) was designed to provide three components: (1) a training module for naïve operators; (2) main routine which operates the processing module and performs data acquisition; (3) data viewer with signal processing algorithm to differentiate positive from negative samples. A Samsung Galaxy S3 (Samsung Electronics, Suwon, South Korea) was used as the mobile device for this study.

The software code for the microcontroller was written using the Arduino platform (Arduino, Italy). The software design utilizes a finite state machine (FSM) architecture with each step of the assay managed as separate states within the code. PID control for thermal incubation modules was programmed and calibrated using an embedded thermistor inside the heating block, and the temperature offset was measured by monitoring liquid temperature directly using an external thermistor (Fig. S3).

### Design and fabrication of mobiNAAT droplet magnetofluidic cartridge

The mobiNAAT cartridge was fabricated by assembling four layers of PMMA sheets (McMaster-Carr, Elmhurst, IL, USA) as outlined in Fig. S4. The chamber layer was laminated with PTFE tape (McMaster-Carr, Elmhurst, IL, USA) on one side in order to render the surface hydrophobic. The sheet was subsequently cut and laminated with the upper layer to generate hydrophilic PMMA chambers. The lower layer was laminated with PTFE film and engraved in order to expose the PMMA surface along the perimeter for bonding. A spacer frame was lined on both faces with acrylic pressure-sensitive adhesive film (3 M Company, St. Paul, MN, USA) to provide a watertight seal and spacing between the upper and lower layers. The top layer was pre-assembled with chamber and spacer layers to form a single component, loaded with assay reagents and bonded with the lower layer. The cartridge was then flipped right side up and filled with approximately 100 μL FC-40 fluorinated oil. Afterwards, a mixture of 30 μL binding buffer and 4 μL magnetic particles was loaded into the sample chamber and sealed at the inlet port with plastic tape prior to transportation. Prior to each assay, sample lysate was loaded into the sample chamber of the cartridge using a disposable pipette.

### Droplet cartridge processing routine

Principles of particle manipulation operations, including particle extraction and washing, are presented and characterized in Fig. S5. For the extraction efficiency study, a modified version of the cartridge with inlets on every chamber was used to facilitate retrieval of droplet from the cartridge. For each measurement, 100 μg magnetic particles were used in 30 uL of PCR-grade water. Rotor was programmed to move from eclipsed position at angular speeds ranging from 2.4–212°/s. Rotor speed was controlled using code developed with the Servo library on an Arduino microcontroller board, where the rotor was actuated in approximately 0.1 degree increments using HS-485HB servo (Hitec RCD, Poway, CA, USA). Angular speed was derived from the duration of motion that was measured in a video capture of rotor motion. Changing the time interval between each increment enabled controlled variation in rotor speed during this study. Residual particles were quantitated by absorbance measurement at 400 nm using NanoDrop 2000 (Thermo Fisher Scientific, Waltham, MA, USA). For the purpose of taking photography illustrating droplet operations, 100 μg magnetic particles and food dyes of varying colors expressed in deionized water were used. Particle washing was characterized by introducing the organic dye fluorescein into the sample inlet and quantifying the presence of fluorescein at the reaction chamber. Each washing step added an aliquot of 25 μL wash buffer between the sample inlet and the reaction chamber. A decrease in fluorescence intensity indicates removal of carryover dye at each particle extraction step. Fluorescence was measured in the FAM emission setting using NanoDrop 3000 fluorometer (Thermo Fisher Scientific, Waltham, MA, USA).

### Image processing

The data acquisition routine was performed by the mobile app and camera as described in the Results section. Photographs were taken at a resolution of 3264 × 2448 over a 60-minute incubation period and were normalized by subtracting the first image of the sequence. For each image acquired, a single vector of green component in RGB space along the center line of the image was extracted. After baseline subtraction, area under the curve (AUC) was calculated by integrating all elements of the vector in the region of interest corresponding to the reaction chamber. In the real-time measurement experiment described in Fig. 3b, time-lapse images were taken using an Android app (Lapse-It) at a resolution of 640 × 480. In the analysis of blinded samples as shown in Fig. 3d, photographs were taken at the beginning and at the end of a 30-minute incubation period. Threshold AUC fluorescence for sample classification was established using a training set of 20 positive and negative samples. Positive controls were prepared by spiking 10^4^ copies of synthetic DNA target into the sample mixture, and the targets were replaced with PCR-grade distilled water in negative controls. Threshold was established at 5 standard deviations above negative control AUC fluorescence (Fig. S6).

For the samples processed using the portable prototype at the emergency room, the positivity algorithm was modified to assess the time integral of signal in a 10-minute window rather than the endpoint fluorescence (Fig. S7). Using this approach, rapid signal development due to amplification event could be distinguished clearly from low levels of signal developed in negative samples. Raw signal was processed in a similar approach as described in Fig. 3a, except the analysis region was extended to the entire 2-dimensional image rather than a single vector. Threshold AUC × Δt value for sample classification was established at 5 standard deviations above negative control AUC × Δt value using a training set of 15 cartridges with negative control as input (Fig. S9).

### Droplet magnetofluidic LAMP assay design

Valid target sequences for primer design were first identified using the NCBI GenBank database (http://www.ncbi.nlm.nih.gov/genbank/) and were checked for cross-reactivity with other organisms prior to primer design. Using PrimerExplorer V4 (Fujitsu Ltd., Japan), several primer sets were designed against various targets in the CT genome and were tested for threshold time and absolute signal difference from baseline (Fig. S10). Based on the results, a primer set designed to target a 321-bp region located in the CT ompA gene was selected for subsequent experiments. All primers used for this experiment are presented in Table S2.

LAMP reaction mixture consisted of the following: primer set (0.2 µM F3/B3, 0.2 µM FIP/BIP, 0.8 µM LF/LB), 0.8 M betaine (Sigma-Aldrich, St. Louis, MO, USA), 1 × Isothermal Amplification Reagent, 6 mM MgSO4, 8 U Bst 2.0 WarmStart DNA Polymerase (New England Biolabs, Ipswich, MA, USA), 1 × LoopAmp Fluorescent Reagent (EIKEN Corp., Japan) and 1.4 mM dNTP set (Life Technologies, Carlsbad, CA, USA). During assay sensitivity characterization, we were able to observe identical results from two sets of complexometric indicator dyes, namely calcein and hydroxynaphthol blue (HNB). HNB can potentially reduce device cost by obviating the need for optical filters required for fluorescent indicators such as calcein, but we observed that the limited stability of HNB in solution affected the consistency of signal across experiments. Calcein generated consistent results over time and was selected as the indicator for the assay in subsequent experiments. In quantitative LAMP characterization experiments, the indicator dye was replaced with 1 × EvaGreen DNA binding dye (Biotium, Inc., Hayward, CA, USA). Primers and synthetic targets were synthesized by Integrated DNA Technologies (Coralville, IA, USA).

LAMP reaction was then characterized for temperature sensitivity (Fig. S11) and for the effect of sample preparation reagents on amplification (Fig. S12). For each reaction, 10 μL synthetic target solution was mixed with input reagent composed of 50 μL lysis buffer, 10 μL resuspension buffer, 4 μL magnetic particles and 30 μL binding buffer. Each washing step used 25 μL wash buffer, followed by incubation in a 25 μL amplification mixture.

### DNA retrieval characterization as function of sample pH

Performance of DNA capture system as a function of sample pH was characterized as follows. 1 × PBS solution at a starting pH of 7.4 (Quality Biological, Gaithersburg, MD, USA) was titrated to prepare buffers at pH 3, 4, 5, 6, 7 and 8 (±0.2) using 0.1 M NaOH and 1 M HCl. For each pH, 50 μL of pH buffer was added to a mixture containing 180 μL lysis buffer, 20 μL magnetic particles and 20 μL lambda DNA stock (New England Biolabs, Ipswich, MA, USA). The mixture was divided into three 70 μL aliquots, and 30 μL binding buffer was added to each aliquot. The particles were subsequently washed twice with 100 μL wash buffer and eluted in 25 μL elution buffer for 5 minutes. Each eluent was quantitated by absorbance measurement at 260 nm using NanoDrop 2000. Retrieved DNA as a function of pH is presented in Fig. S13.

### Clinical sample testing conditions

Vaginal swab samples were collected from patients and archived in −80 °C before use. The swab was expressed by gently dabbing the cotton tip against a 600 μL microcentrifuge tube containing ~200 μL lysis reagent composed of 50:20:1 lysis buffer, resuspension buffer and proteinase K solution by volume (ChargeSwitch gDNA mini bacteria kit, Life Technologies). Afterwards, the tube was inserted into an on-board thermal lysis chamber and incubated for 30 minutes at 65 °C followed by 15 minutes at 95 °C for proteinase inactivation. Swab samples tested at the emergency room were similarly expressed in lysis buffer without proteinase K and lysed using a portable microbead-beating unit (OmniLyse Kit, Claremont BioSciences). 70 μL of the lysate was transferred to the sample inlet of the droplet cartridge.

### Emergency department study design

Institutional review board (IRB) application was prepared as an amendment to an ongoing clinical impact assessment study for commercially available rapid tests for *Chlamydia trachomatis*, *Neisseria Gonorrhoea* and *Trichomonas vaginalis*. All experimental protocols were approved by the Johns Hopkins Medicine Institutional Review Board, and all methods were carried out in accordance with relevant guidelines and regulations. Subjects were pre-screened for all eligibility and exclusion criteria, including a discussion with the treating provider to ensure the patient’s plan of care during the ED visit includes STI testing. Informed consent was obtained from all subjects. After this initial screening and consent, patients were fully enrolled in the study and randomized to either the control group or the rapid testing group. For all subjects enrolled in the control group, two vaginal swabs were collected by the provider during the pelvic exam. For all subjects enrolled in the rapid testing group, three vaginal swabs were collected by the provider during the pelvic exam. For both groups, one swab was used for conventional testing of chlamydia and gonorrhea at the clinical laboratory, while the other swab was used for evaluation in the emergency room using the mobiNAAT platform. The results obtained from the mobiNAAT platform were withheld from the provider as the device is not FDA-cleared. The remaining swab from the rapid testing group was used for evaluation in a commercial rapid testing platform located in the clinical laboratory.

## Electronic supplementary material

Supplementary Video S1

Supplementary Figures
