# Supplementary material for: Mobile nucleic acid amplification testing (mobiNAAT) for Chlamydia trachomatis screening in hospital emergency department settings
Source: Sci Rep. 2017 Jul 3;7:4495. doi: 10.1038/s41598-017-04781-8 (PMC5495747; doi:10.1038/s41598-017-04781-8)
Supplement: Supplementary file 2 — Supplementary Figures [file 41598_2017_4781_MOESM2_ESM.doc]

Title: Mobile nucleic acid amplification testing (mobiNAAT) for Chlamydia trachomatis screening in hospital emergency department settings

**Authors:** D. J. Shin^1^, P. Athamanolap^1^, L. Chen^2^, J. Hardick^3^, M. Lewis^4^, Y. H. Hsieh^4^, R. E. Rothman^34^, C. A. Gaydos^34^, T. H. Wang*^125^

**Affiliations:**

^1^Department of Biomedical Engineering, The Johns Hopkins University, Baltimore, MD, 21218, USA.

^2^Department of Mechanical Engineering, The Johns Hopkins University, Baltimore, MD, 21218, USA.

^3^Division of Infectious Diseases, School of Medicine, The Johns Hopkins University, Baltimore, MD, 21218, USA.

^4^Department of Emergency Medicine, School of Medicine, The Johns Hopkins University, Baltimore, MD, 21218, USA.

^5^Institute for NanoBioTechnology, The Johns Hopkins University, Baltimore, MD, 21218, USA.

*To whom correspondence should be addressed: Professor Tza-Huei Wang; Present address: 118B Clark Hall, 3400 N Charles Street, Baltimore, MD 21218, USA; Phone: +1-410-516-7086; E-mail: thwang@jhu.edu

**Supplementary Materials**

Fig. S1. Sensitivity and specificity panels for calcein-LAMP reagent

Fig. S2. Mobile phone user interface

Fig. S3. Thermal incubator calibration

Fig. S4. Magnetofluidic droplet cartridge fabrication workflow

Fig. S5. Particle manipulation mechanism and characterization

Fig. S6. Control samples for threshold setting in clinical validation

Fig. S7. Positivity algorithm based on time derivative signal

Fig. S8. Clinical NAAT instrument RLU data

Fig. S9. Negative controls for threshold setting in emergency room testing

Fig. S10. LAMP primer performance evaluation

Fig. S11. LAMP operating temperature characterization

Fig. S12. Inhibitor characterization

Fig. S13. DNA retrieval as function of input buffer pH

Table S1. Complete bill of materials and cost breakdown in US$ (2015)

Table S2. Primer sequences designed for amplification mixture

Video S1. User training modules


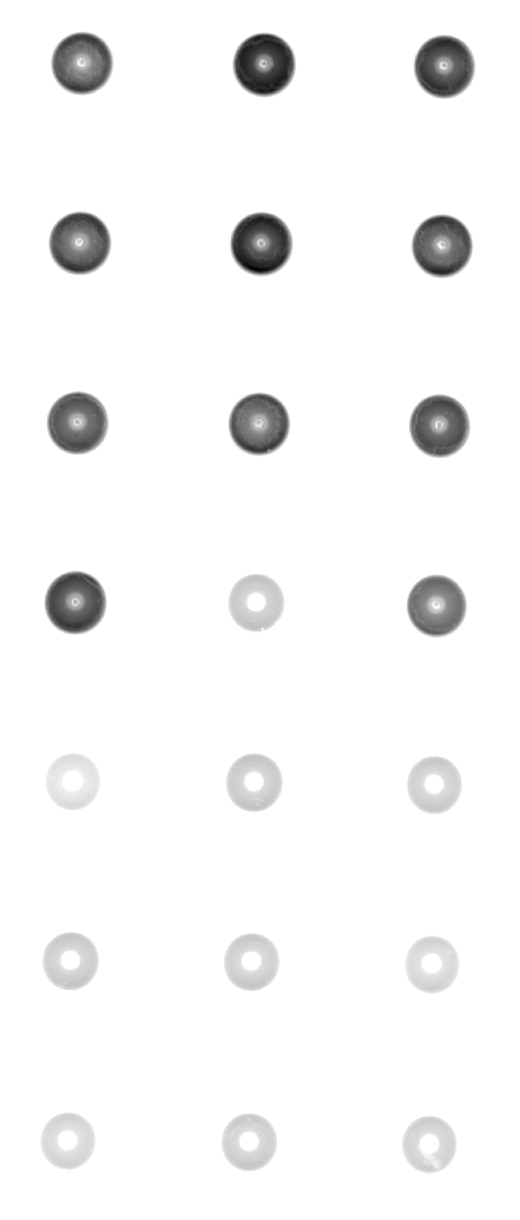


**10^5^ copies**

**10^4^ copies**

**10^3^ copies**

**10^2^ copies**

**10^1^ copies**

**10^0^ copies**

**Water**

**a**

**
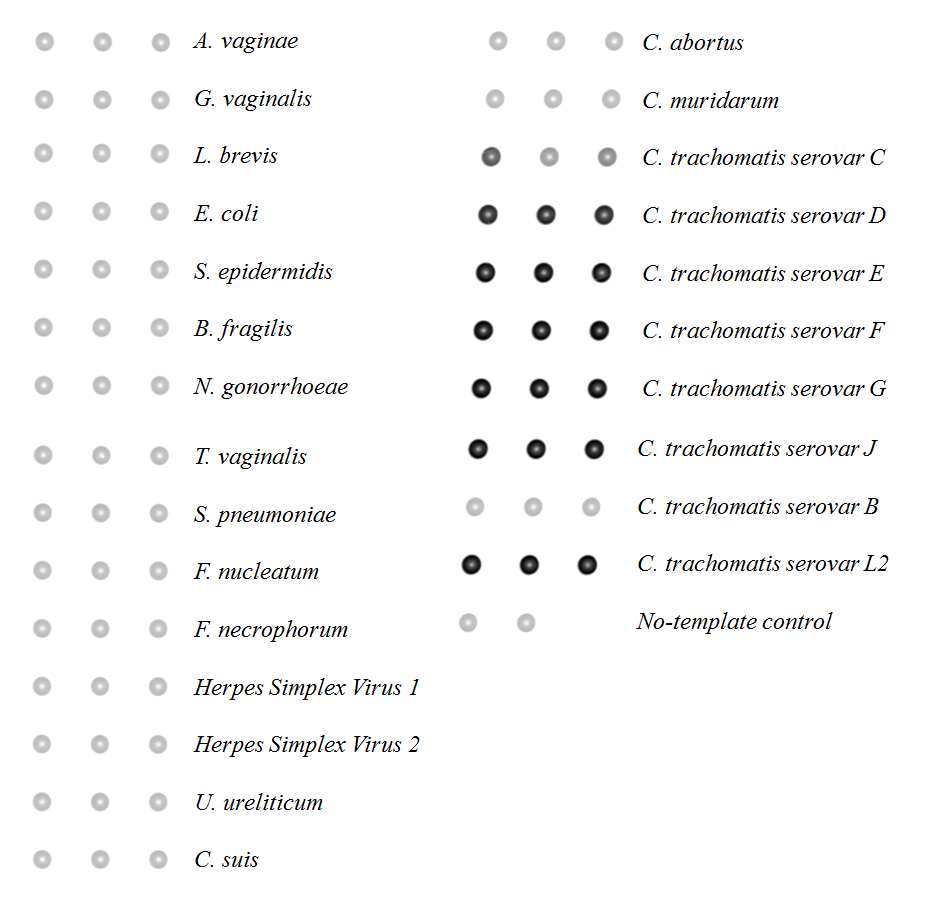
**

**b**

**Fig. S1.** Sensitivity and specificity panels for calcein-LAMP reagent. (**a**) Sensitivity of the LAMP assay tested using calcein indicator dye at various concentrations of synthetic target. Up to 10^3^ copies of molecular target could be consistently amplified with observable difference. (**b**) Specificity of the LAMP assay tested using calcein indicator dye using genomic DNA extracts from endogenous and pathogenic vaginal flora. Genomic DNA was purified from single strains of each species included in the specificity panel using an automated DNA purification system (MagNA Pure LC, Roche Diagnostics). All vaginal Chlamydia trachomatis serovars amplified correctly; serovars B and C are relevant to ocular infection and will not be present in vaginal swabs. All genomic DNA samples tested were prepared at concentrations exceeding or equal to 10^5^ copies of molecular target per mL.

**a**

**
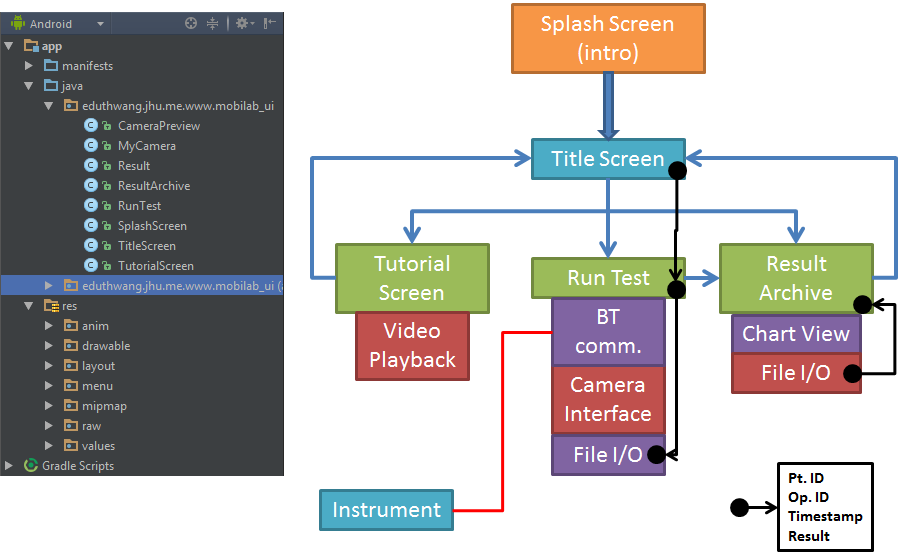
**

**b**


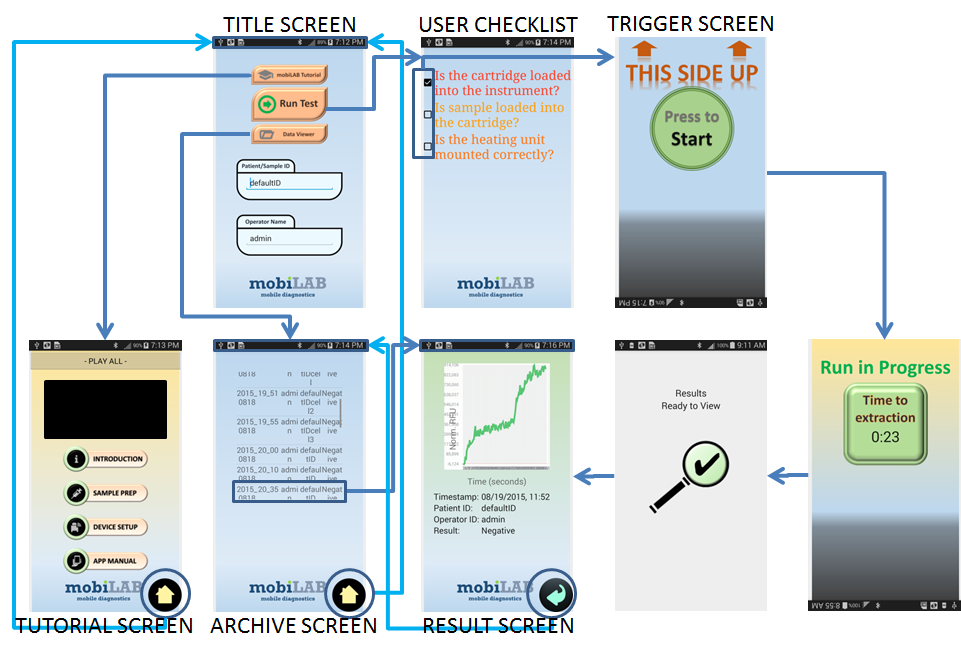


**Fig. S2.** Mobile phone user interface. (**a**) UI architecture. The UI consists of a central title screen, which allows the user to access three options: 1) a tutorial screen, which facilitates playback of training videos; 2) a run screen, which runs the assay; 3) a result archive, which provides access to results obtained from previous runs; (**b**) Screenshot of the mobile phone user interface at various stages of assay progression. Title Screen enables initiation of the three routines through the on-screen buttons and provides input fields for recording sample and operator ID. In the main routine, the checklist ensures that the users complete steps external to the mobile app before the processing module is engaged. Once the checklist is completed, the app transitions to a trigger screen that engages the processing module at the press of a button. After each routine is accessed, the user can return to the Title Screen by clicking on the Home or Return buttons.


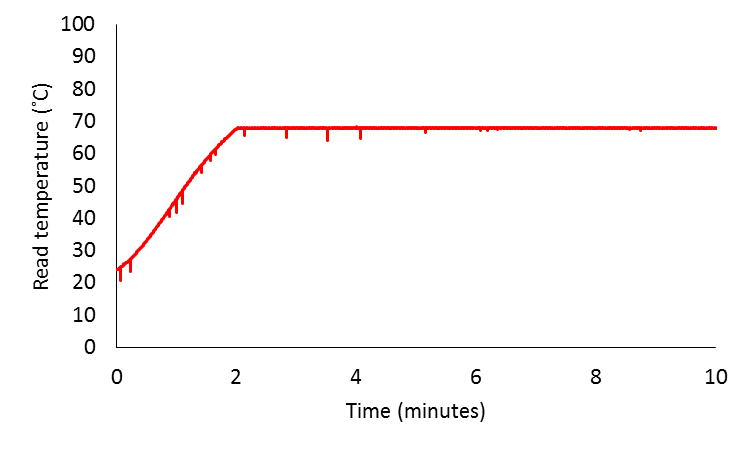


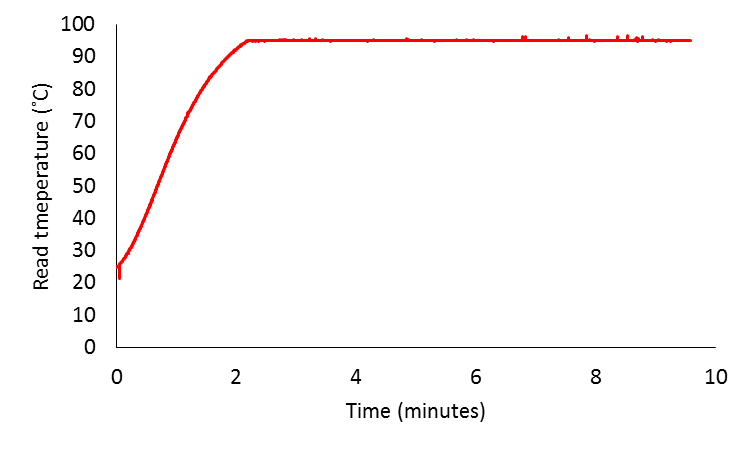


**a**

**b**

**Fig. S3.** Thermal incubator calibration. Each temperature was calibrated using the following method: First, PID parameters (Kp, Kd and Ki) were modified until overshoot and oscillation were no longer observed with a rise time of approximately 2 minutes. Afterwards, a test device with a thermistor in contact with the heated liquid was placed in contact with the incubator. Target temperature was modified until steady state target temperature could be achieved by the heated liquid. (**a**) Temperature profile for 65˚C incubation. With target temperature at 66 ˚C, the PID controller yielded a steady-state temperature reading at 68˚C, which corresponded to liquid temperature of 65~67˚C. (**b**) Temperature profile for 95˚C incubation. With target temperature at 93 ˚C, the PID controller yielded a steady-state temperature reading at 95˚C, which corresponded to a liquid temperature of 95~98˚C.

**
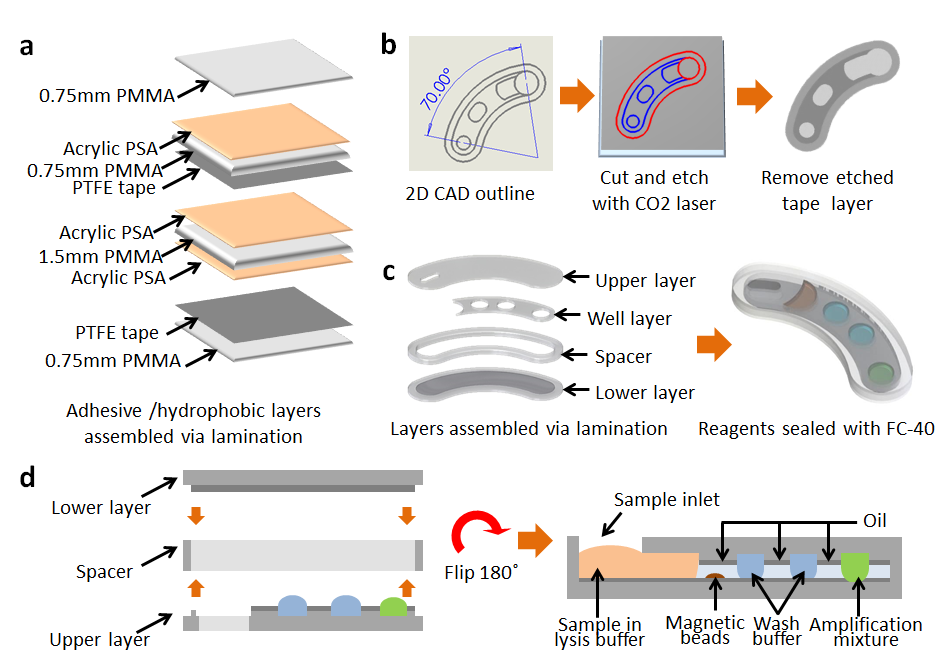
**

**Fig. S4.** Magnetofluidic droplet cartridge fabrication workflow. (**a**) Device is prepared on a layer-by-layer basis. Upper and lower layers are generated by coating the surface of a 0.75-mm PMMA sheet with PTFE tape. The spacer frame between two layers is generated by coating a 1.5-mm PMMA sheet on both surfaces with acrylic pressure-sensitive adhesive (PSA). The well layer is similarly generated using a 0.75-mm PMMA sheet. (**b**) CAD software was used to generate laser paths for the CO_2_ laser cutter, operating in both cutting and engraving modes. The engraving mode allowed cutting of the PTFE film only, which could be selectively peeled away in order to reveal a hydrophilic surface that could be used to anchor aqueous reagents or to provide bonding surface for acrylic PSA. (**c**) Layer assembly results in a sealed droplet cartridge. (**d**) Detailed description of layer assembly. Reagents were primed on an upside-down upper layer, followed by assembly of the spacer and lower layer via compression. Flipping the entire cartridge right side up and filling it with fluorinated oil result in an assay-ready cartridge.


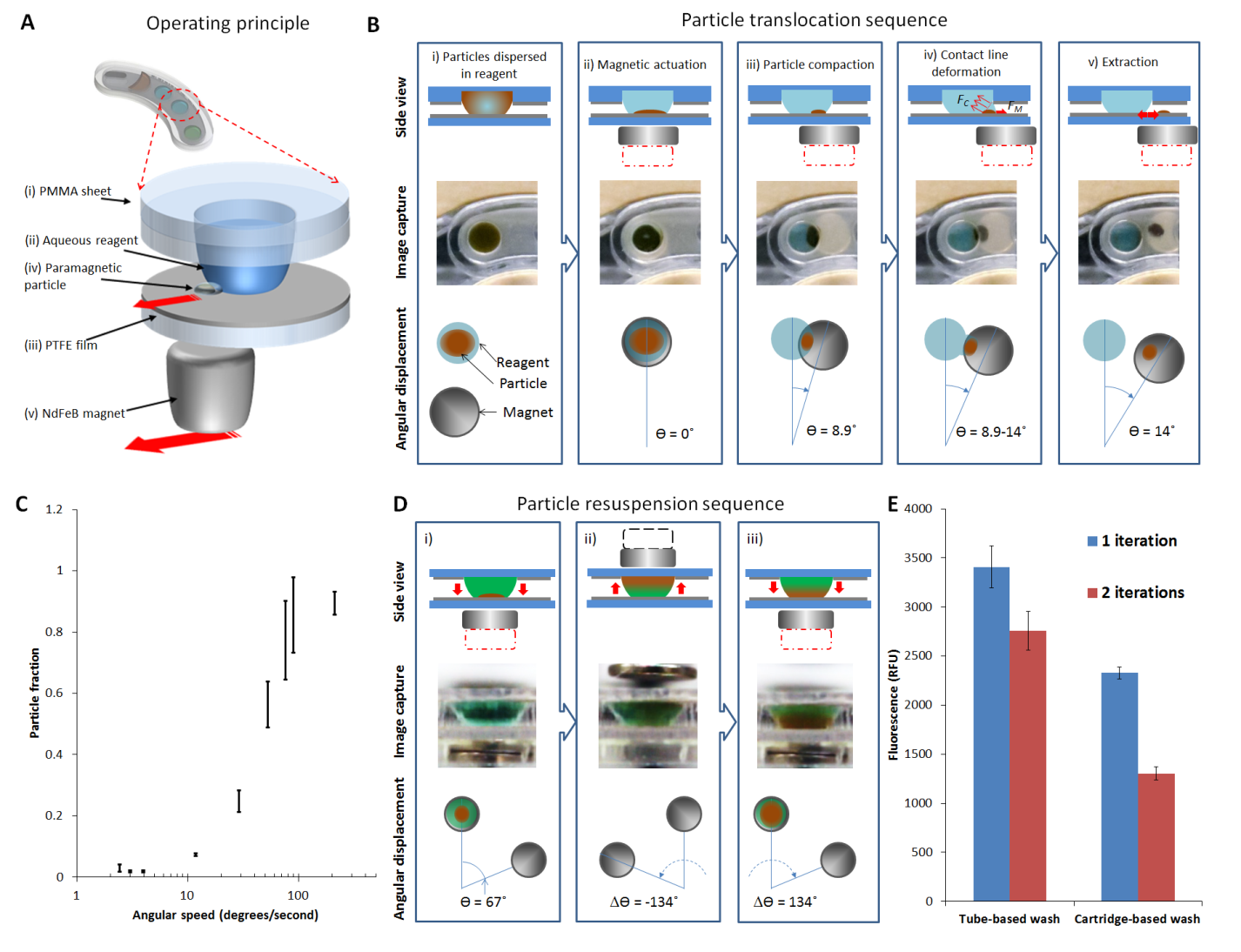


**Fig. S5.** Particle manipulation mechanism. (**A**) Principle of droplet magnetofluidic manipulation. Aqueous reagents (ii) are immobilized on open-top chambers fabricated using hydrophilic substrates such as poly (methyl methacrylate) (PMMA) (i). The reagents form a non-wetting liquid column upon contact with a hydrophobic surface such as polytetrafluoroethylene (PTFE) (iii), which enables translocation of paramagnetic particles (iv) on the plane when acted upon by a time-varying magnetic field generated by a moving permanent magnet (v). (**B**) Diagram describing the particle translocation mechanism. As the magnetic particles are concentrated into a plug (iii) and actuated by a moving permanent magnet, droplet kinematics is reduced to interaction of two forces: the force generated by magnetic attraction of particles towards the permanent magnet (F_M_) and the capillary force (F_C_) formed around the deformed fluid interface as the plug approaches the reservoir’s periphery (iv). When F_M_> F_C_, particles are liberated from the reservoir (v). (**C**) Particle dissociation characteristics as a function of actuator speed. Particle fraction indicates the amount of residual particle inside the droplet. Particle extraction achieved maximal efficiency at angular speeds below 10˚/s and complete disengagement from the actuator at angular speeds above 100˚/s. (**D**) Diagram describing the washing mechanism. In order to facilitate efficient rinsing of magnetic particles and reduce the amount of aqueous reagent carried over with the particle plug at each step of the assay, the processing module utilized two magnetic spokes for mixing. Top and bottom spokes alternate (sequences i🡪iii) in order to facilitate transversal motion of the magnetic particles. The magnetic field pulls the particles across from one surface to the opposite surface (red block arrows), facilitating disintegration of the particle plug into a plume as magnets alternate position. (**E**) Characterization of fluorescein washing in a standard 1.5 mL microcentrifuge tube in a bench process and on the cartridge using particle extraction and resuspension.

**c**


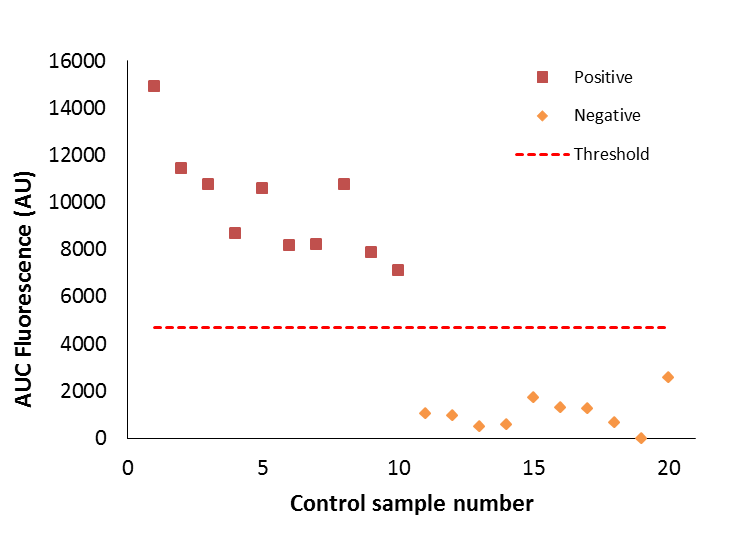


**Fig. S6.** Control samples for threshold setting in clinical validation. Ten positive and ten negative samples were used as a training set to establish threshold AUC fluorescence for classification. Threshold was initially defined as the average fluorescence level from negative samples + 5σ and verified to be sufficiently stringent to differentiate between positive and negative samples in the testing set.


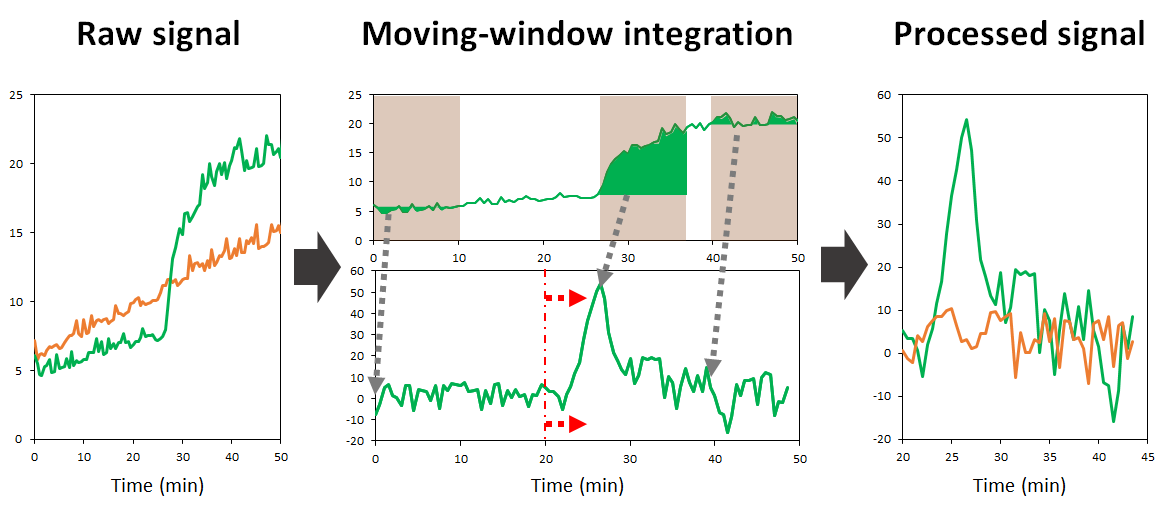


**Fig. S7.** Positivity algorithm based on time derivative signal. Positivity algorithm on the field-tested prototype utilizes moving-window integration in order to detect sharp transitions in fluorescence observed during amplification. Although endpoint detection based on raw signal can suffice in a controlled environment, the differences between positive and negative samples may become obscured due to factors including ambient lighting condition and low levels of background fluorescence development during thermal incubation. In order to exclude irregular signal obtained during early stages of amplification from data analysis, a time threshold is applied to process data after 20 minutes of incubation. As a means to identify rapid polymerization events indicative of LAMP amplification, a 10-minute moving window was applied to the data. As seen in the processed signal, this algorithm acts as a high-pass filter that accentuates contrast between positive (green line) and negative (orange line) reactions.


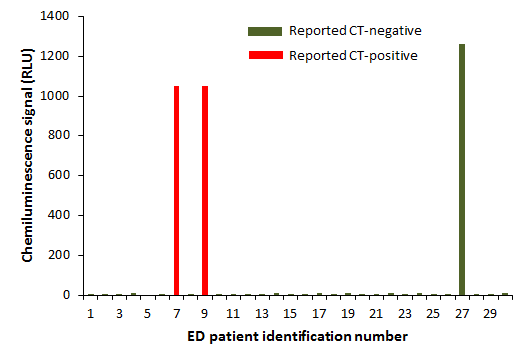


**Fig. S8.** Raw instrument data obtained from clinical NAAT control tests for samples collected at the emergency department. Horizontal axis represents sample ID and vertical axis represents chemiluminescence from the signaling probe in relative luminescence units (RLU). The Gen-Probe Aptima Combo 2 assay is a duplex assay which differentiates between CT and NG-positive signals from the same optical channel based on the kinetic profiles of the chlamydia and gonorrhea probes. Samples 7 and 9 which were CT-positive and NG-negative, and results are in concordance with mobiNAAT data. As the RLU data does not distinguish between chlamydia and gonorrhea, the Gen-Probe assay also shows high RLU for sample 27 (which was CT-negative and NG-positive). These results were fully concordant with a third test run using a commercial PCR-based testing platform (Cepheid GeneXpert, Sunnyvale, CA, USA).


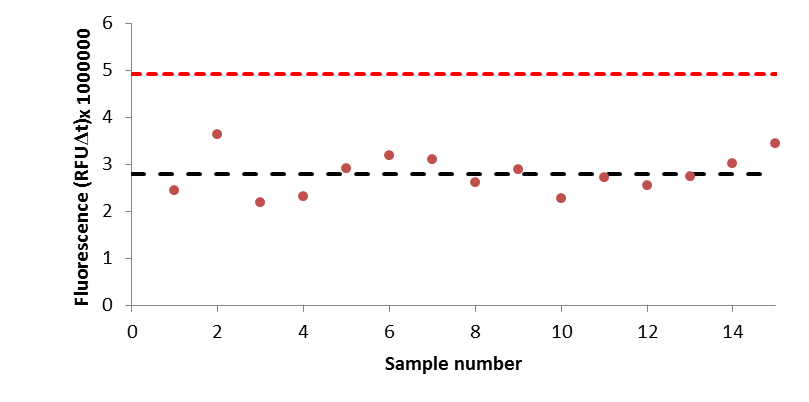


**Fig. S9.** Negative controls for threshold setting in emergency room testing. Negative controls for field test in the emergency room. Threshold (red dashed) was set 5 standard deviations above average (black dashed).


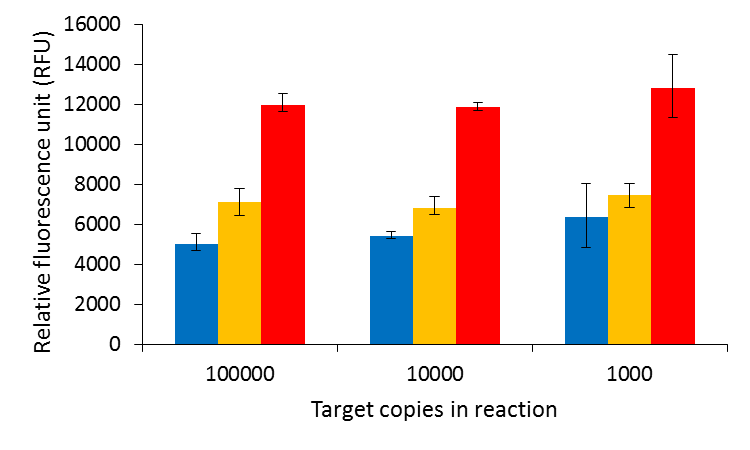


**A**

**b**


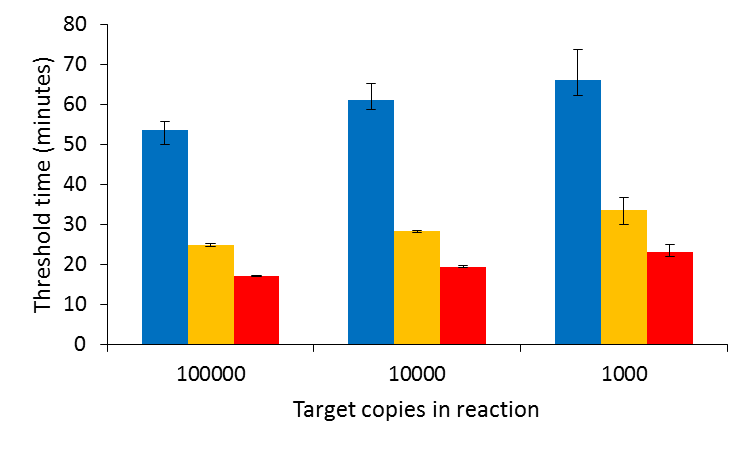


**a**

**Fig. S10.** LAMP primer performance evaluation. (**a**) Absolute baseline-subtracted signal obtained by three different LAMP primer sets with targets in three orders of magnitude dilution in relative fluorescence unit (RFU). Primer set 3 (red) demonstrates approximately twofold higher signal than either primer set 1 (blue) or 2 (orange). (**b**) Threshold time obtained by the same primer sets. Primer set 3 (red) demonstrates earlier amplification of signal above threshold than either primer set 1 (blue) or 2 (orange).

**Fig. S11.** LAMP operating temperature characterization. In order to assess the level of stringency required to maintain adequate isothermal amplification conditions, temperature was varied between 56˚C and 74˚C, and its effect on time threshold was observed. Based on real-time signals, we identified an optimal window of approximately 4˚C between 64.5˚C and 68.5˚C. Input DNA was set at 1,000 copies of target per reaction. This window was subsequently used as the basis for thermal control on the mobiNAAT platform.

**
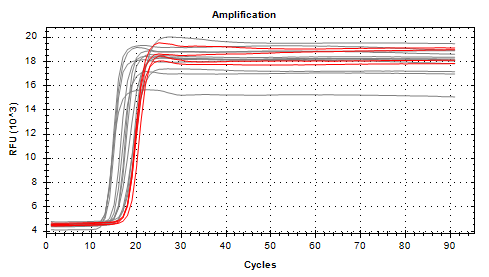

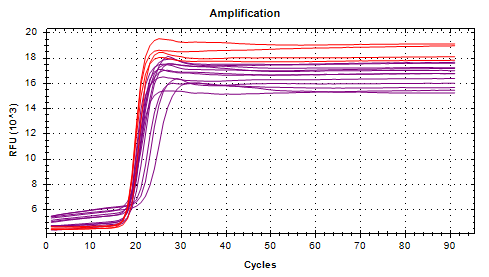
**

**a**

**b**

**
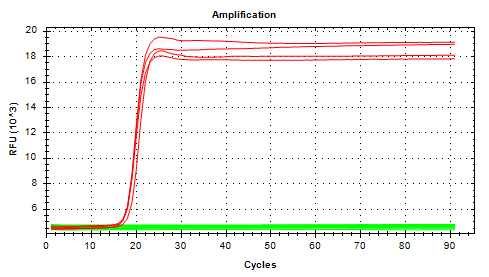

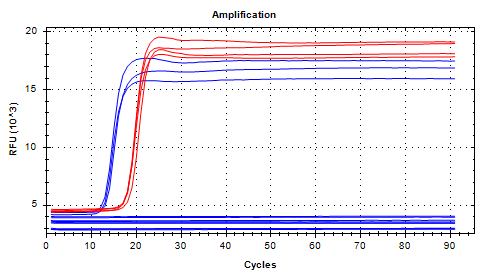

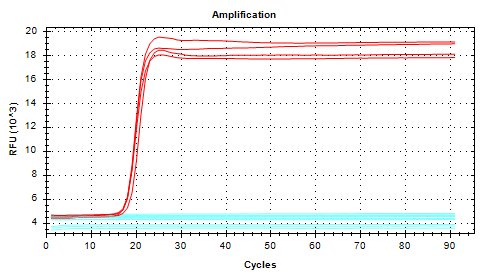
**

**c**

**d**

**e**

**Fig. S12.** Inhibitor characterization. For each of the reagents utilized in DNA purification, a small amount of reagent was spiked into the amplification mixture containing 10^3^ targets and incubated. In all traces, red line indicates positive control reaction. (**a**) Cell culture (positive control). (**b**) Lysis buffer. Amplification was not significantly affected. (**c**) Binding buffer. This reagent has substantially acidic pH, which may account for most reactions failing. (**d**) Proteinase K. All reactions containing proteinase K failed to amplify. (**e**) A mixture containing lysis buffer, proteinase K, and binding buffer. The results indicate that thorough bead washing and thermal deactivation of proteinase K are important parameters for the single-stream assay to perform optimally.

**
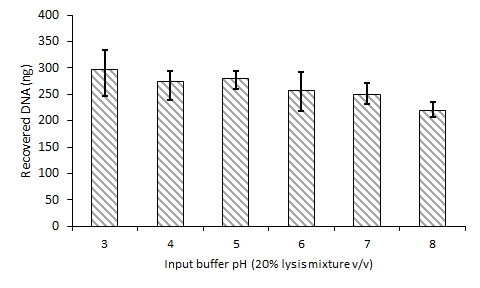
**

**Fig. S13.** DNA retrieval as function of input buffer pH. 200 μL lysis buffer containing lambda DNA and magnetic particles was mixed with 50 μL buffered pH solutions at ranges 3-8 to mimic vaginal swab . 50 μL is considered a reasonable estimate of eluent volume from swab according to Warnke et al (http://dx.doi.org/10.1371/journal.pone.0102215). For the pH ranges between 3 and 5, variance across pH was smaller than variance within replicates. There is a gradual decrease in DNA recovery with an increase in pH, with a sharper decrease at transition between pH 7 to pH 8 (12.7% decrease). Considering that typical vaginal pH rests between 3.5 and 4.5, this data supports the relevance of electrostatic DNA recovery system in vaginal sample matrix.

**Table S1.** Complete bill of materials and cost breakdown in US$

**Table S2.** Primer sequences designed for amplification mixture

Primer set 1: Targeting 16S rRNA gene in *Chlamydia trachomatis* genome

| **Primer** | **Sequence** |
| --- | --- |
| **F3** | CGTTAGTTGCCAGCACTTA |
| **B3** | ACACGCCATTACTAGCAATT |
| **LIP** | CATAAGGGCCATGCTGACTTGAAACTCTAACGAGACTGCCT |
| **BIP** | TACAGAAGGTGGCAAGATCGCCAGACTACAATCCGAACTGG |
| **LF** | TCGCCTTCCTCCTGGTTA |
| **LB** | GATGGAGCAAATCCTCAAAGC |

Primer set 2: Targeting 16S rRNA gene in *Chlamydia trachomatis* genome

| **Primer** | **Sequence** |
| --- | --- |
| **F3** | GATGCAACGCGAAGGACC |
| **B3** | TGGTTAACCCAGGCAGTCT |
| **LIP** | GACAGCCATGCAGCACCTGTTGTATATGACCGCGGCAGAA |
| **BIP** | GTGCCGTGAGGTGTTGGGTTAACACCCTAAGTGCTGGCAAC |
| **LF** | GTCCTTGCGGAAAACGACA |
| **LB** | ACGAGCGCAACCCTTATCG |

Primer set 3: Targeting ompA gene in *Chlamydia trachomatis* genome

| **Primer** | **Sequence** |
| --- | --- |
| **F3** | GTTCTGCTTCCTCCTTGC |
| **B3** | TGGAATTCTTTATTCACATCTGT |
| **LIP** | GATCTCCGCCGAAACCTTCCGGAATCCTGCTGAACCAA |
| **BIP** | TTGCGATCCTTGCACCACTTCGGTCGAAAACAAAGTCAC |
| **LF** | CAGAATTCCGTCGATCATAAGGC |
| **LB** | TGACGCTATCAGCATGCGTATG |

**Video S1.** User training modules

Instructions are segmented in a chronological fashion, outlining a general overview of the platform followed by an instruction on manual steps outside the mobile app. The tutorial concludes with a walkthrough on how to navigate the mobile app to perform the assay. The entire training module is approximately 6 minutes in length.
